# A Tellurium Oxide Microcavity Resonator Sensor Integrated On-Chip with a Silicon Waveguide

**DOI:** 10.3390/s18114061

**Published:** 2018-11-21

**Authors:** Henry C. Frankis, Daniel Su, Dawson B. Bonneville, Jonathan D. B. Bradley

**Affiliations:** Department of Engineering Physics, McMaster University, 1280 Main Street West, Hamilton, ON L8S 4L7, Canada; sux11@mcmaster.ca (D.S.); bonnevd@mcmaster.ca (D.B.B.); jbradley@mcmaster.ca (J.D.B.B.)

**Keywords:** photonic sensors, optical microcavities, resonators, silicon photonics

## Abstract

We report on thermal and evanescent field sensing from a tellurium oxide optical microcavity resonator on a silicon photonics platform. The on-chip resonator structure is fabricated using silicon-photonics-compatible processing steps and consists of a silicon-on-insulator waveguide next to a circular trench that is coated in a tellurium oxide film. We characterize the device’s sensitivity by both changing the temperature and coating water over the chip and measuring the corresponding shift in the cavity resonance wavelength for different tellurium oxide film thicknesses. We obtain a thermal sensitivity of up to 47 pm/°C and a limit of detection of 2.2 × 10^−3^ RIU for a device with an evanescent field sensitivity of 10.6 nm/RIU. These results demonstrate a promising approach to integrating tellurium oxide and other novel microcavity materials into silicon microphotonic circuits for new sensing applications.

## 1. Introduction

Modern health diagnostics and environmental sensing are benefitting from the emergence of various lab-on-chip integrated optical devices that enable compact, cheap, sensitive, and rapid assessment. In particular, the silicon-on-insulator (SOI) platform has been shown to provide mass producible, low optical loss components with compatibility for biological sensing, chemical detection, and temperature monitoring [1,2,3,4,5,6,7,8,9,10,11,12]. Compared to alternative electrical techniques, these on-chip optical sensors benefit from electromagnetic insensitivity, rapid assessment over a wide bandwidth, and low-cost. The material systems utilize high refractive index contrast waveguides, including silicon on silica and silicon nitride on silica, which can experience tight bends for the realization of compact optical circuits from the visible through to mid-infrared sensing windows. Various devices have been investigated in terms of their sensitivity and performance as optical sensors including Mach–Zehnder interferometers [13], photonic crystals [1,4,5,12], and microring and microdisk resonators [1,2,3,4,5,6,7,8,9,10,11]. Of these devices, important figures of merit include size, strong light–matter interaction between the mode and sensing medium, sensitivity and selectivity of measurement events, and ease of data collection. Due to its sharp and selective resonance spectra along with compact size, the ring resonator is an ideal sensor for the SOI integrated optical platform.

Ring resonators integrated on silicon for sensing purposes have been demonstrated in a variety of materials including silicon [1,2,3,4,5,6,7,8,9,10,11,12], polymers [14,15], silicon oxynitride [16], and silicon nitride [17,18,19], which are the materials typically available in silicon photonics foundries. As silicon sensors begin to approach their fundamental limitations [5] new directions of research must be studied. Access to new waveguiding materials on the SOI platform could allow for the design and fabrication of sensors with improved *Q* factors for more precise sensing, larger evanescent fields to improve sensitivity, improved surface bonding chemistry for molecular detection, and the integration of new active components. Here, we explore the sensing capabilities of an on-chip tellurium oxide (TeO_2_) microcavity resonator. Tellurium oxide has been shown to be a low loss optical waveguide material [20], making it ideal for fabricating high *Q* resonators for high resolution sensing [21,22,23,24]. Additionally, TeO_2_ is a suitable host for rare earth ions such as erbium [25], which could allow for laser-based sensing [26,27,28,29]. On-chip microlaser sensors have been demonstrated using Yb:SiO_2_ and Er:SiO_2_, in which the laser resonators are toroid structures and their ultra-narrow emission lines can be used to detect single nanoparticles in air or water [30,31]. Although highly sensitive and effective, these devices required the external coupling of the laser output to an off-chip optical fiber. Alternatively, coupling the laser output directly to an on-chip waveguide might enable a more robust form factor and the integration of such sensors within silicon microphotonic circuits.

In this work, we fabricate and characterize a TeO_2_ microcavity resonator sensor coupled to a silicon waveguide, giving access to tellurium oxide’s material advantages in a structure that is compatible with silicon waveguide technology and processing. The device was fabricated in a standard wafer-scale silicon photonics foundry. Pre-defined circular trenches were etched into the chip’s SiO_2_ top cladding layer followed by post-processing TeO_2_ film deposition, similar to the process flow used for the fabrication of aluminum oxide rare earth lasers on a silicon nitride platform [32,33]. The cavity described in this work allows for the realization of the first mass producible TeO_2_ sensor integrated into the SOI platform with the potential to produce active laser-based sensing cavities. The approach described here can be applied more broadly to realize a wide variety of microcavity sensor materials on an SOI platform.

## 2. Microcavity Properties and Characterization

The sensor chips were fabricated using the Advanced Micro Foundry (AMF) silicon photonics fabrication process. Silicon bus waveguides of 0.5 μm width were patterned into a 0.22-μm-thick silicon layer on a 2 μm buried oxide on silicon-on-insulator wafers. A 3-μm-thick SiO_2_ top cladding was then deposited over the bus waveguides, after which 10-µm-wide and 80-µm-outer-diameter circular trenches were etched into the cladding. The edge of each circular trench was aligned to the edge of a bus waveguide, with a nominal gap of 0.2 µm. A deep etch was then carried out to form end facets, and the wafer was diced into chips and transferred from the foundry. We then coated the chips in a tellurium oxide layer using a reactive sputtering process similar to [20] as an end of line processing step [34]. The TeO_2_ coating in the trench acts as a waveguiding layer and forms a resonant microcavity, aligned to the silicon bus waveguide by the high-resolution lithography process. The bus waveguides were each tapered to 0.18 μm width at the edge facets of the chip to improve fiber-to-chip coupling efficiency and tapered to a width of 0.35 μm next to the microcavity to aid the bus waveguide to resonant cavity evanescent coupling. The reduced waveguide width caused the waveguide mode’s evanescent field to expand, leading to better overlap with the resonant mode of the microcavity. It also reduced the effective index of the waveguide mode to 2.05, which matched closely to the refractive index of tellurium oxide (2.07 [20]) for better phase matching. To further improve the coupling efficiency, the bus waveguide was wrapped around the cavity resonator in a pulley-coupled design with a 42 μm length.

**Figure 1:**
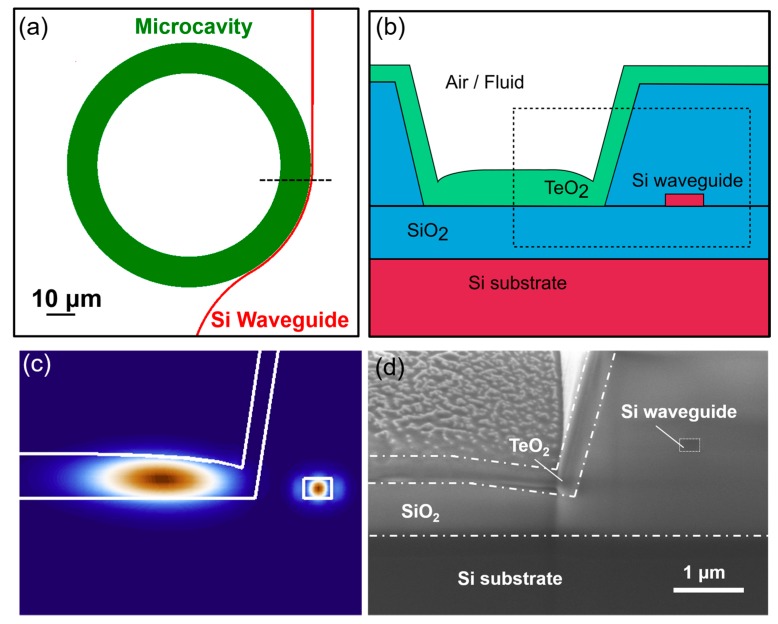
(**a**) Top view drawing of the resonator sensor showing the pulley-coupled silicon bus waveguide (red) and the TeO_2_ microcavity (green). (**b**) Cross-section schematic of the device through the section indicated by the dashed line in (**a**), showing the silicon bus waveguide and the TeO_2_ resonator layer coated into the trench. (**c**) Calculated fundamental transverse electric (TE) polarized electric field mode profiles for the TeO_2_ resonator and silicon bus waveguide in the region indicated by the dashed line in (**b**). (**d**) Focused ion beam (FIB) scanning electron microscope (SEM) cross-section image of a fabricated device showing the realized structure.

The device structure is shown in Figure 1. Figure 1a shows a top view drawing microcavity design, including the TeO_2_ resonator and the pulley-coupled silicon bus waveguide. Figure 1b displays a cross sectional schematic of the designed waveguide and resonant structure. For the sensing experiments, we coupled the signal light from a fiber into the silicon bus waveguide, where it was routed towards the resonator. We then monitored changes in the light transmitted through the bus waveguide. Figure 1c shows the fundamental transverse electric (TE) mode profiles supported by both the bus waveguide and the resonator, as calculated by a finite element eigenmode solver. Figure 1d shows a focused ion beam (FIB) milled scanning electron microscope (SEM) cross-section image of a fabricated device. From the image it is evident that the SiO_2_ trench was etched below the level of the buried oxide, and a lateral offset was introduced by the sidewall angle of the trench etch. The vertical and lateral offset of the fabricated trench structure resulted in a significantly larger gap between the resonator and waveguide compared to the designed gap of 0.2 μm, reducing the optical overlap between the bus waveguide and cavity resonator modes for less efficient evanescent coupling. This reduction in coupling efficiency was compensated for by the pulley-coupled design.

**Figure 2:**
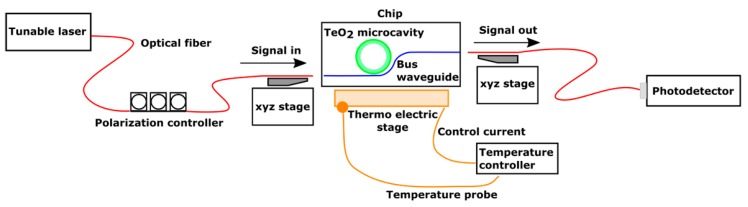
Diagram of the optical setup used to characterize the devices.

We characterized the devices using an optical edge-coupling setup, shown in Figure 2. Light from a tunable laser, with a range of 1510 to 1640 nm and down to 0.1 pm resolution, was guided by an optical fiber through polarization-controlling paddles to a cleaved fiber. The cleaved fiber was aligned to the bus waveguide using an xyz translation stage. The polarization paddles were adjusted to couple light to the TE polarized waveguide mode. A second cleaved fiber was aligned on the output facet to collect the transmitted signal and coupled to a photodetector. Transmission spectra for the devices were collected by sweeping the wavelength of the tunable laser and measuring the corresponding transmitted power at the photodetector at each wavelength. 

For this work we fabricated three chips that were coated in 480-nm-, 900-nm- and 1100-nm-thick tellurium oxide films for testing. The chips underwent initial passive measurements to characterize the resonators. Typical figures used to characterize the resonators include the extinction ratio (ER) or how much the transmitted signal decreases when on a resonant wavelength, the free spectral range (FSR) or the periodic spacing between resonant wavelengths and the *Q* factor, which is inversely proportional to the spectral width of the resonance dip and the cavity loss. The 480-nm-thick TeO_2_ coated chip was found to have resonances with a 2.8 dB extinction ratio, *Q* factors of up to 0.4 × 10^5^, corresponding to an 8.7 dB/cm waveguide loss and FSRs of 4.5 nm at 1510, increasing to 5.1 nm at 1600 nm. The 900-nm-thick coated chip had resonances with a larger 9 dB extinction ratio, *Q* factors of up to 0.7 × 10^5^, corresponding to a 5.1 dB/cm waveguide loss and FSRs of 5.0 to 5.2 nm with resonances occurring between the 1597 and 1628 nm wavelengths. The 1100-nm-thick coated chip had resonances with a 3.2-dB extinction ratio, larger *Q* factors of up to 1.6 × 10^5^, corresponding to a 2.2 dB/cm waveguide loss and FSRs of 4.9 to 5.1 nm, with resonances occurring between the wavelengths 1592 and 1628 nm. The 2.2 dB/cm loss in the 1100-nm-thick TeO_2_ resonator is comparable to a standard SOI waveguide and is approaching the background loss threshold that would be necessary to achieve lasing, based on an ~3 dB/cm net gain demonstrated in erbium-doped TeO_2_ waveguides [25]. The three microcavity devices were used to perform thermal and evanescent field sensing experiments.

## 3. Sensing Experiments

### 3.1. Thermal Sensing

We measured the devices using the transmission setup shown in Figure 2. The chips were mounted on a thermoelectric Peltier cooler stage. A temperature feedback controller uses a temperature probe to monitor the temperature of the stage and adjust the current applied to the Peltier cooler to achieve a desired temperature setpoint. To measure the thermal shift, the stage was initially set at 20 °C and the resonance spectrum was measured. The temperature setpoint was then increased in increments of 5 °C up to 40 °C. At each increment, the stage was left at the temperature setpoint for 5 min. to allow for the chip to reach thermal equilibrium with the stage. The tunable laser wavelength was then swept to identify resonant wavelengths.

**Figure 3:**
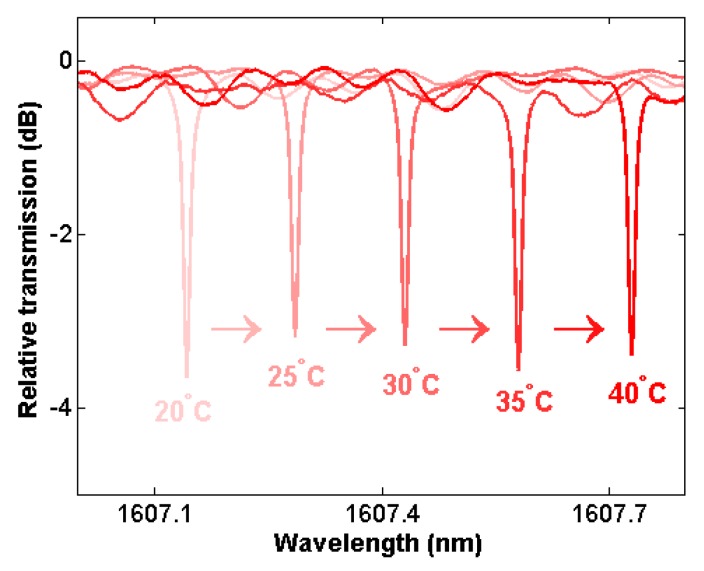
Resonance spectra measured at temperatures ranging from 20 to 40 °C for the microcavity with a 1100-nm-thick TeO_2_ film, showing shifting of the resonance wavelength.

As the temperature of the device changes, the cavity experiences the effects of thermal expansion and temperature-dependent refractive index changes (the thermo-optic effect), changing the electric field profile of the propagation mode and its optical path length. The change to the optical path length results in a shift to the resonance wavelength of the cavity. Figure 3 shows the optical spectrum of a cavity from 1607 to 1608 nm measured at each temperature. The resonance wavelength is seen to shift from 1607.14 nm at 20 °C to 1607.73 nm at 40 °C, with approximately equal wavelength shifts at each temperature interval.

**Figure 4:**
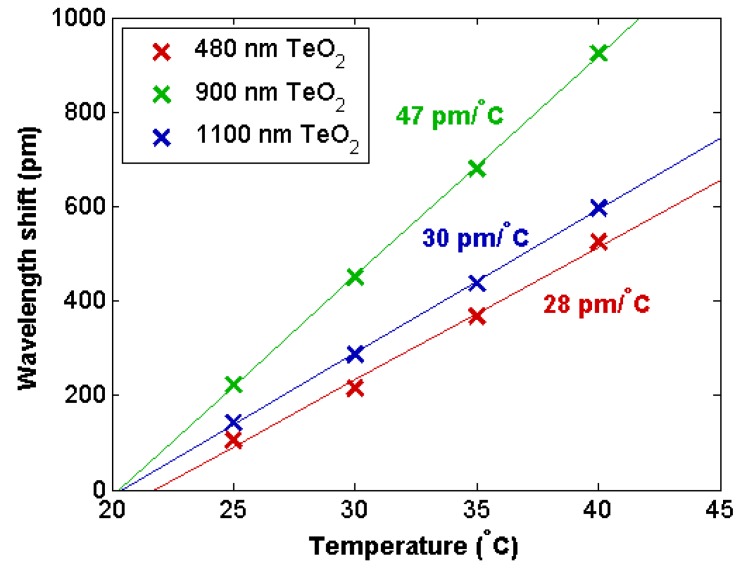
Measured wavelength shift versus temperature for 480-, 900-, and 1100-nm-thick TeO_2_ cavities, fitted to have thermal sensitivities of 28, 47, and 30 pm/°C, respectively.

We measured the thermally-induced resonance shift for the resonant cavities with 480-, 900- and 1100-nm-thick TeO_2_ films. The resonant wavelength shift at each temperature interval was recorded relative to the resonant wavelength at 20 °C. The wavelength shift versus temperature was fit with a line to extract the resonators’ thermal sensitivity in wavelength per °C, as displayed in Figure 4. All cavities demonstrated a linear relationship with a correlation coefficient greater than 0.99. The 480-, 900- and 1100-nm-thick TeO_2_ cavities were found to have thermal sensitivities of 28, 47, and 30 pm/°C, respectively. At a TeO_2_ thickness of 480 nm finite element waveguide mode simulations calculated the power confinement of the resonator mode in the tellurium oxide layer to be approximately 80%, while the 900-nm- and 1100-nm-thick TeO_2_ samples had very similar confinements of approximately 90%. Since all three resonators had similar optical confinements in the tellurium oxide layer, the thermo-optically-related temperature shift can be expected to be similar among all devices. The observed differences in the thermal sensitivities might be explained by variations in film quality, slight differences in confinement and optical intensity in the resonator, and variations due to thermal expansion, for example in the thinnest sample, where the waveguide mode interacts the most with the sidewall. Using the thermal shift data, an estimation of tellurium oxide’s thermo-optic coefficient can be made by assuming that the thermal shift is completely determined by the thermo-optic effect. Using this assumption, the thermo-optic coefficient (*σ*_T_) of tellurium oxide is estimated from the thermal sensitivity (∆*λ*/∆*T*), cavity mode group index (*n*_g_) and resonant wavelength (*λ*_0_). A sample calculation for the 1100-nm-thick TeO_2_ resonator, with a 30 pm/°C sensitivity at a 1602.2 nm wavelength, with a group index of 2.05 based on a 4.97 nm FSR is shown below:

(1) $$\begin{array}{ll} \sigma_{T} & =\frac{\Delta \lambda }{\Delta T}\cdot \frac{n_{g}}{\lambda_{0}}\\ & =30\frac{\mathrm{pm}}{{}^{{}^{\circ}}C}\cdot \frac{2.05}{1602.2\;\mathrm{nm}}\\ & =3.8\times 10^{-5}{/}^{{}^{\circ}}C \end{array}$$

Similar calculations for the 480-nm- and 900-nm-thick coated samples gave thermal sensitivities of 3.6 × 10^−5^ and 5.9 × 10^−5^/°C, respectively. This range of thermo optic coefficients was approximately 3–5 times the thermo optic coefficient of silica, and 0.3–0.5 times the thermo-optic coefficient of silicon, and agreed reasonably well with previous values reported for tellurite glass [35].

Comparatively, thermal sensing in silicon waveguides has been shown to reach sensitivities of 83 pm/°C [7], approximately two to three times the sensitivity demonstrated here. Because silicon has a much larger thermo-optic coefficient than tellurium oxide and changing the film thickness of the resonator has been shown to have a minimal effect the thermal sensitivity of the device, it is unlikely that a significantly higher sensitivity can be achieved. Pathways to higher thermal sensitivities would likely require using a material with a larger thermo optic coefficient than tellurium oxide in the cavity structure. However, a low thermal sensitivity can also be advantageous for greater temperature stability during other types of sensing measurements.

### 3.2. Evanescent Field Sensing

Biological sensing using the SOI platform is typically based on evanescent field sensing, where changes to the refractive index in the region of a waveguide’s evanescent field change the effective index of the waveguide mode, which can be measured as a shift in the resonance wavelength of a resonant device. Microfluidic channels fabricated above the optical resonator can be used for the detection of biological markers in the fluid. For this experiment, the evanescent field sensing capabilities of the resonator were characterized by measuring the resonant wavelength shift after coating the chip in deionized (DI) water using the optical setup shown in Figure 2. The temperature stage was set to approximately room temperature at 20 °C. A reference resonance spectrum was first measured in air by sweeping the tunable laser with 0.1 pm step size. The chip was then covered in a layer of DI water. The water was assumed to also be at room temperature such that it would not cause a large thermal shift in the resonance wavelength. The optical spectrum was then remeasured and compared to the reference spectrum.

**Figure 5:**
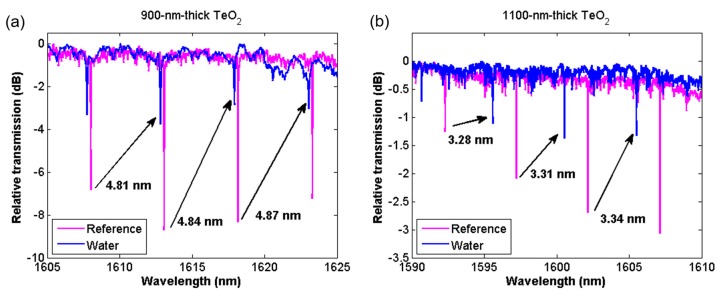
Resonance spectra measured in air (pink) and after covering the chip in water (blue) for (**a**) 900-nm-thick and (**b**) 1100-nm-thick TeO_2_ resonators.

Figure 5 shows a comparison of the resonance spectra for the 900-nm- and 1100-nm-thick TeO_2_ microcavities before and after water was coated over the chip. Both spectra clearly show shifting resonance wavelengths. In Figure 5a it can be seen that the resonances of the water-coated sample have almost begun to shift past one FSR in the 900-nm-thick resonator. The 900-nm-thick TeO_2_ microcavity experienced a shift of 4.81 nm for an initial resonance at 1608.00 nm, increasing to a shift of 4.87 nm for the resonance wavelength at 1618.16 nm. Figure 5b shows that the 1100-nm-thick TeO_2_ microcavity experienced wavelength shifts ranging from 3.28 to 3.34 nm. As seen in the results, longer wavelengths and thinner TeO_2_ films lead to larger resonance shifts by extending the waveguide mode’s evanescent tail further into the water coated on top of the resonator, thereby increasing device sensitivity.

**Figure 6:**
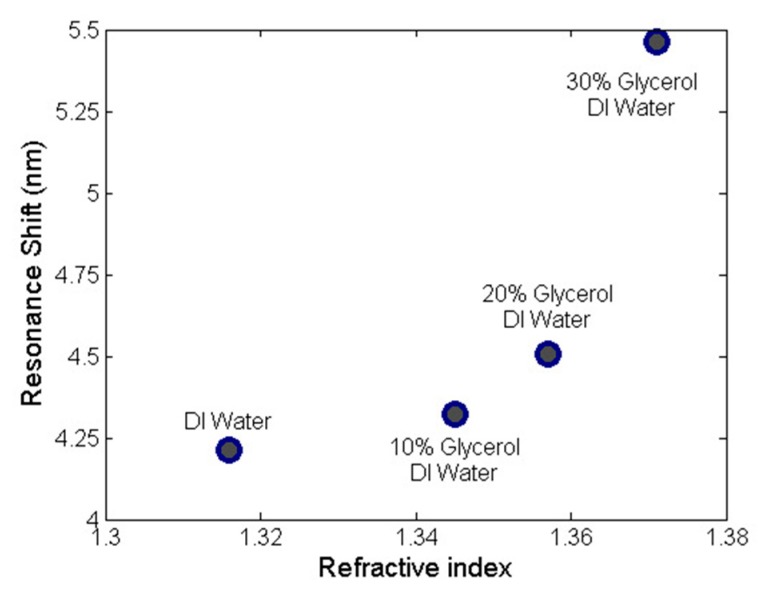
Measured resonance shift vs. cladding refractive index for a 900-nm-thick TeO_2_ microcavity coated in solutions with varying concentrations of glycerol and water.

Taking the refractive index of water as 1.316, and the refractive index of the original air cladding as 1, the refractive index unit (RIU) sensitivity of the 900-nm-thick coated device was measured to be:

(2) $$\frac{\Delta \lambda }{\Delta n_{\mathrm{cladding}}}=\frac{4.87}{1.316-1}=15.4\frac{\mathrm{nm}}{\mathrm{RIU}},$$

where Δ*λ* is the resonant wavelength shift and Δ*n*_cladding_ is the change in cladding refractive index. A similar calculation carried out for the 1100-nm-thick coated sample based on the 3.34 nm resonant wavelength shift at 1592 nm, which gave a device sensitivity of 10.6 nm/RIU. We also characterized the resonant shift in the 900-nm-thick TeO_2_ microcavity as a function of the glycerol concentration in water to characterize the local sensitivity for indices ranging from 1.316–1.371. The measured shifts are plotted in Figure 6, demonstrating a local sensitivity of 19.6 ± 1.3.

**Figure 7:**
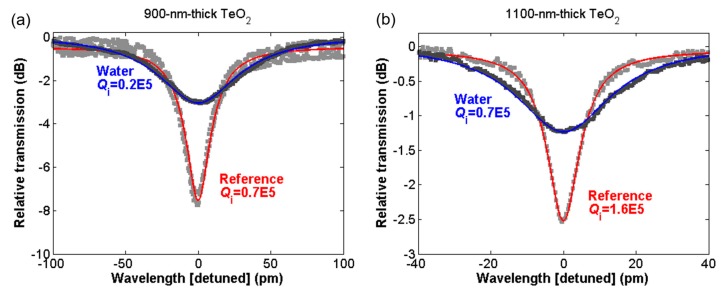
Cavity resonance modes around 1600 nm measured in air (fit with red line) and after coating the chip in water (fit with blue line) for (**a**) 900-nm-thick and (**b**) 1100-nm-thick TeO_2_ resonators, demonstrating resonance broadening in water.

Along with shifting the resonance wavelength, the addition of water was also seen to result in a decrease in the *Q* factor of the resonator. This is a result of water’s absorption around 1550 nm, which caused optical attenuation in the cavity. Figure 7a compares the *Q* factor fit for the 900-nm-thick TeO_2_ resonator before and after its being coated in water, demonstrating a large broadening of the resonance. The intrinsic *Q* factor was observed to decrease from 0.7 × 10^5^ to 0.2 × 10^5^, representing an additional 12.9 dB/cm of waveguide loss. Figure 7b similarly compares the *Q* factor of a resonant mode before and after the addition of water for the 1100-nm-thick TeO_2_ resonator. The *Q* factor decreased from 1.6 × 10^5^ down to 0.7 × 10^5^, corresponding to an additional 2.9 dB/cm of loss. The smaller increase in attenuation in the thicker resonator was caused by a decreased optical overlap with the water. When the evanescent field sensing of the 480-nm-thick TeO_2_ device was tested, the resonance modes were seen to disappear completely after the application of water, as the loss became too great, extinguishing the resonances.

We used a finite element mode simulation to model the evanescent field sensitivity of devices. The waveguide modes for resonators with 500-, 700-, 900-, and 1100-nm-thick TeO_2_ layers were simulated at cladding refractive indices ranging from air (*n*_cladding_ = 1) to 1.5 in steps of 0.01. At each step the effective index (*n*_eff_) of the simulated waveguide mode was recorded. The expected resonant wavelength shift (Δ*λ*) for the device was then calculated using the change in effective index (∆*n*_eff_) relative to the simulated effective index for an air cladding (*n*_eff0_) for an initial resonant wavelength (*λ*_0_), using the equation:

(3) $$\Delta \lambda =(\Delta n_{\mathrm{eff}})\frac{\lambda_{0}}{n_{\mathrm{eff}0}}.$$

**Figure 8:**
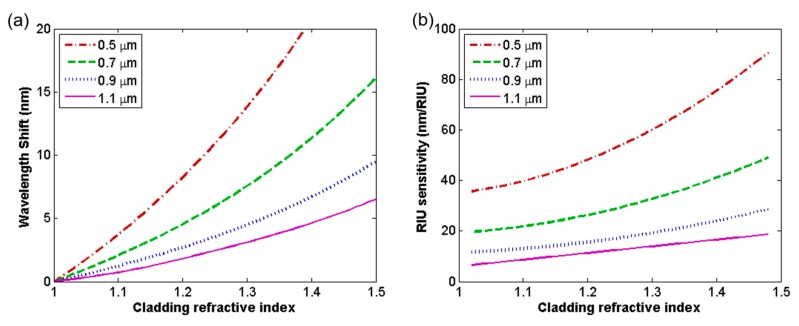
Simulated (**a**) wavelength shift and (**b**) RIU sensitivity vs. evanescent medium refractive index for cavities with TeO_2_ coating thicknesses ranging from 0.5 to 1.1 μm.

Figure 8a shows the predicted wavelength shift for different cladding refractive indices calculated for different TeO_2_ layer thicknesses at a wavelength of 1600 nm. Simulations for the 900-nm-thick TeO_2_ device predicted a wavelength shift of 4.81 nm for a DI water cladding (*n*_cladding_ = 1.316), which agreed well with the measured 4.81 nm shift. Likewise, the 1100-nm-thick TeO_2_ device’s simulated 3.32 nm wavelength shift agreed well with the measured 3.34 nm shift. Based on this simulation, the 480-nm-thick TeO_2_ device could have been expected to have a shift of approximately 14.8 nm if its resonances were not extinguished. It can also be seen from the figure that the wavelength shift versus cladding refractive index is not a linear relationship, with greater sensitivity as the cladding refractive index increases. This relationship is a result of the decreased refractive index contrast between the TeO_2_ waveguide and the cladding at larger cladding refractive indices, causing the evanescent tail of the waveguide mode to leak more into the cladding. This effect becomes more pronounced in the TeO_2_ microcavity than it would be in a silicon waveguide, where the refractive index of the silicon waveguide core is significantly larger than the cladding medium. This effect results in a discrepancy between the measured data based on Figure 5 and the simulated RIU sensitivity, which is shown in Figure 8b. The measured RIU sensitivity assumed a perfectly linear shift with the refractive index. However, because the true relationship was nonlinear, the actual local RIU sensitivity of the device around the cladding index of water was larger. Based on the simulations, the actual local sensitivity of the 900-nm-thick coated sample was 19.9 nm/RIU, which is in reasonable agreement with the data shown in Figure 6, while the 1100-nm-thick coated sample had a local sensitivity of 14.3 nm/RIU.

The sensitivities demonstrated here of 15.4 and 10.6 nm/RIU are relatively low compared to the state of the art in SOI evanescent field sensitivity, with 247 and 248 nm/RIU sensitivity demonstrated using microring resonators [4] and photonic crystal resonators [12], respectively, for example. Based on the simulations shown in Figure 8, the sensitivity of the microcavity resonators could be improved by using devices with thinner TeO_2_ coatings. However, to achieve high sensitivities a large evanescent field overlap with water is needed. Greater overlap with water leads to greater water absorption, which will reduce the *Q* factor of the resonator, as shown in Figure 7. Lower *Q* factors widen the bandwidth of the resonator, making it more difficult to distinguish wavelength shifts. A sensor’s limit of detection (LOD) [36], which is the change in RIU needed to the shift the resonant wavelength of a resonator by one bandwidth when coated in water, is a metric used to account for this effect. The LOD is determined by the sensing wavelength (λ), *Q* factor of the device while submerged in water (*Q*), and the evanescent field sensitivity of the device (*S*). Ideally, devices will have low LODs. The following shows an example calculation for the 900-nm-thick TeO_2_ resonator, which was found to have a LOD of 5.2 × 10^−3^ RIU:

(4) $$\begin{array}{ll} \mathrm{LOD} & =\frac{\lambda }{Q\cdot S}\\ & =\frac{\left(1600\;\mathrm{nm}\right)}{(20000)\cdot \left(15.4\frac{\mathrm{nm}}{\mathrm{RIU}}\right)}\\ & =5.2\times 10^{-3}\mathrm{RIU} \end{array}$$

Similarly, we calculated a LOD of 2.2 × 10^−3^ for the 1100-nm-thick TeO_2_ device. This demonstrates that despite the thinner film’s larger sensitivity, its ability to distinguish RIU shifts is lower. Similarly, when comparing these results to the high sensitivity microring resonator sensor (LOD of 3.3 × 10^−3^), and photonic crystal resonator sensor (LOD of 5.2 × 10^−3^), their limit of detection was found to be larger than the LOD demonstrated in the 1100-nm-thick TeO_2_ resonator. Considering sensors with low LOD, rather than just high sensitivity, such as the microring resonator sensor of [37] with a 2.9 × 10^−4^ limit of detection, the LOD demonstrated here is an order of magnitude higher than the state of the art in evanescent field detection.

## 4. Discussion

We demonstrated an on-chip tellurite glass resonator sensor that is integrated with a silicon bus waveguide and fabricated using a silicon-photonics-compatible process flow. Three chips each coated in a different thickness of TeO_2_ were used to characterize the thermal and evanescent field sensing capabilities of the resonator, with a list of figures of merit for each device summarized in Table 1. The devices with thicker TeO_2_ films were found to offer higher performance in terms of sensing. The thicker resonators had a larger extinction ratio, allowing for a better signal to noise ratio, had a larger *Q* factor for greater sensing resolution, a larger thermal sensitivity, and were able to perform evanescent field sensing without overly degrading the quality factor of the resonator.

As silicon-based sensors have begun to approach their fundamental detection limit [5], imposed by the effect of water-absorption-limiting *Q* factors, directions in integrated optical sensor research will move towards systems level integration and new device functionality. A dielectric-based sensor such as TeO_2_ is rare earth soluble, enabling the potential fabrication of a rare-earth-doped laser sensor, which would not be possible using a purely SOI sensor, since silicon is not rare earth soluble. The cavity structure used here allows for the dielectric sensor to be coupled to a silicon bus waveguide, which can enable monolithic integration of the sensor into larger scale silicon photonic systems. A similar cavity structure using an aluminum oxide film coupled to a silicon nitride bus waveguide has demonstrated both high *Q* factors (>1 × 10^6^) [38] and rare earth lasing [32,33], suggesting that improved limits of detection and the successful implementation of laser sensors are possible.

Future research on this sensing platform could focus on the full calibration of the evanescent field sensor and implementation of a microfluidic delivery system for biological marker identification. Due to tellurium oxide’s solubility in certain solutions [39] a more practical device design would use a thin silicon dioxide cap layer to prevent the direct contact of sensing fluids with the TeO_2_. Investigating alternate sensing methods, such as spectroscopic sensing, where analytes coated onto the resonator can be fingerprinted by their absorption lines and their resulting effect on a thermal shift of the resonator is also measured [40], is of interest. Fabricating sensor cavities with larger ring radii, different film thicknesses, a thin SiO_2_ or polymer cap layer to passivate surface roughness and using an extra HF etch step to smooth the cavity sidewall, could all be used to improve device *Q* factors. Rare earth doping of the tellurite glass can be explored for laser-based sensors and the integration of such micro resonator sensors within silicon photonic circuits, for example on-chip germanium detectors for sensor readout would be of great interest.

## 5. Conclusions

We demonstrated a TeO_2_ microcavity sensor on a silicon photonic platform. The sensor was characterized for thermal sensitivity with a maximum sensitivity of 47 pm/°C. Evanescent field sensing measurements yielded a sensitivity and limit of detection of 10.6 nm/RIU and 2.2 × 10^−3^ RIU, respectively, for an 80-μm-diameter and 1100-nm-thick TeO_2_ microcavity. These results show that this platform is promising for integrated high *Q* and laser sensing devices. Using the approach presented here, we propose that TeO_2_ and other novel resonator materials can be integrated into silicon photonic circuits for new sensing applications.

## Figures and Tables

**Table 1:** Figures of merit for the tested 480-nm-, 900-nm- and 1100-nm-thick TeO_2_ resonators.

Figure of Merit	480-nm-Thick TeO_2_ Resonator	900-nm-Thick TeO_2_ Resonator	1100-nm-Thick TeO_2_ Resonator
Extinction Ratio	2 dB	9 dB	3 dB
Free Spectral Range	5.1 nm	5.1 nm	4.9 nm
Thermal Sensitivity	28 pm/°C	47 pm/°C	30 pm/°C
*Q* Factor (in Air)	0.4 × 10^5^	0.7 × 10^5^	1.6 × 10^5^
*Q* Factor (in Water)	-	0.2 × 10^5^	0.7 × 10^5^
Evanescent Field Sensitivity	-	15.4 nm/RIU	10.6 nm/RIU
Limit of Detection	-	5.2 × 10^−3^ RIU	2.2 × 10^−3^ RIU

## References

[1] Gavela A.F., García D.G., Ramirez J.C., Lechuga L.M. (2016). Last advances in silicon-based optical biosensors. Sensors.

[2] De Vos K., Bartolozzi I., Schacht E., Bienstman P., Baets R. (2007). Silicon-on-Insulator microring resonator for sensitive and label-free biosensing. Opt. Express.

[3] TalebiFard S., Schmidt S., Shi W., Wu W., Jaeger N.A.F., Kwok E., Ratner D.M., Chrostowski L. (2017). Optimized sensitivity of Silicon-on-Insulator (SOI) strip waveguide resonator sensor. Biomed. Opt. Express.

[4] Schmidt S., Flueckiger J., Wu W., Grist S.M., Talebi Fard S., Donzella V., Khumwan P., Thompson E.R., Wang Q., Kulik P. (2014). Improving the performance of silicon photonic rings, disks, and Bragg gratings for use in label-free biosensing. Proc. SPIE.

[5] Chrostowski L., Grist S., Flueckiger J., Shi W., Wang X., Ouellet E., Yun H., Webb M., Nie B., Liang Z. (2012). Silicon photonic resonator sensors and devices. Proc. SPIE.

[6] Iqbal M., Gleeson M.A., Spaugh B., Tybor F., Gunn W.G., Hochberg M., Baehr-Jones T., Bailey R.C., Gunn L.C. (2010). Label-free biosensor arrays based on silicon ring resonators and high-speed optical scanning instrumentation. IEEE J. Sel. Top. Quantum Electron..

[7] Kim G.-D., Lee H.-S., Park C.-H., Lee S.-S., Lim B.T., Bae H.K., Lee W.-G. (2010). Silicon photonic temperature sensor employing a ring resonator manufactured using a standard CMOS process. Opt. Express.

[8] Xu H., Hafezi M., Fan J., Taylor J.M., Strouse G.F., Ahmed Z. (2014). Ultra-sensitive chip-based photonic temperature sensor using ring resonator structures. Opt. Express.

[9] Kim H.-T., Yu M. (2016). Cascaded ring resonator-based temperature sensor with simultaneously enhanced sensitivity and range. Opt. Express.

[10] Janz S., Xu D.-X., Vachon M., Sabourin N., Cheben P., McIntosh H., Ding H., Wang S., Schmid J.H., Delâge A. (2013). Photonic wire biosensor microarray chip and instrumentation with application to serotyping of Escherichia coli isolates. Opt. Express.

[11] Liu J., Zhou X., Qiao Z., Zhang J., Zhang C., Xiang T., Shui L., Shi Y., Liu L. (2014). Integrated optical chemical sensor based on an SOI ring resonator using phase-interrogation. IEEE Photonics J..

[12] Lo S.M., Hu S., Gaur G., Kostoulas Y., Weiss S.M., Fauchet P.M. (2017). Photonic crystal microring resonator for label-free biosensing. Opt. Express.

[13] Guan X., Wang X., Frandsen L.H. (2016). Optical temperature sensor with enhanced sensitivity by employing hybrid waveguides in a silicon Mach-Zehnder interferometer. Opt. Express.

[14] Girault P., Lorrain N., Poffo L., Guendouz M., Lemaitre J., Carré C., Gadonna M., Bosc D., Vignaud G. (2015). Integrated polymer micro-ring resonators for optical sensing applications. J. Appl. Phys..

[15] Zhang D., Men L., Chen Q. (2018). Femtosecond laser fabricated polymer microring resonator for sensing applications. Electron. Lett..

[16] Pucker G., Samusenko A., Ghulinyan M., Pasquardini L., Chalyan T., Guider R., Gandolfi D., Adami A., Lorenzelli L., Pavesi L. (2016). An integrated optical biosensor platform. SPIE Newsroom.

[17] Ksendzov A., Lin Y. (2005). Integrated optics ring-resonator sensors for protein detection. Opt. Lett..

[18] Chen Y., Li Z., Yi H., Zhou Z., Yu J. (2009). Microring resonator for glucose sensing applications. Front. Optoelectron. China.

[19] Wang J., Yao Z., Poon A.W. (2015). Silicon-nitride-based integrated optofluidic biochemical sensors using a coupled-resonator optical waveguide. Front. Mater..

[20] Madden S.J., Vu K.T. (2009). Very low loss reactively ion etched Tellurium Dioxide planar rib waveguides for linear and non-linear optics. Opt. Express.

[21] Guo J., Shaw M.J., Vawter G.A., Hadley G.R., Esherick P., Sullivan C.T. (2005). High-Q microring resonator for biochemical sensors. Proc. SPIE.

[22] Hu J., Sun X., Agarwal A., Kimerling L.C. (2009). Design guidelines for optical resonator biochemical sensors. J. Opt. Soc. Am. B.

[23] Righini G.C., Dumeige Y., Féron P., Ferrari M., Nunzi Conti G., Ristic D., Soria S. (2011). Whispering gallery mode microresonators: Fundamentals and applications. Riv. Nuovo Cimento.

[24] Foreman M.R., Swaim J.D., Vollmer F. (2015). Whispering gallery mode sensors. Adv. Opt. Photonics.

[25] Vu K., Madden S. (2010). Tellurium dioxide Erbium doped planar rib waveguide amplifiers with net gain and 2.8 dB/cm internal gain. Opt. Express.

[26] Peng X., Song F., Jiang S., Peyghambarian N., Kuwata-Gonokami M., Xu L. (2003). Fiber-taper-coupled L-band Er^3+^-doped tellurite glass microsphere laser. Appl. Phys. Lett..

[27] Sasagawa K., Yonezawa Z., Iwai R., Ohta J., Nunoshita M. (2004). S-band Tm^3+^-doped tellurite glass microsphere laser via a cascade process. Appl. Phys. Lett..

[28] Ruan Y., Boyd K., Ji H., Francois A., Ebendorff-Heidepriem H., Munch J., Monro T.M. (2014). Tellurite microspheres for nanoparticle sensing and novel light sources. Opt. Express.

[29] Vanier F., Côte F., El Amraoui M., Messaddeq Y., Peter Y.-A., Rochette M. (2015). Low-threshold lasing at 1975 nm in thulium-doped tellurite glass microspheres. Opt. Lett..

[30] Ostby E.P., Vahala K.J. (2009). Yb-doped glass microcavity laser operation in water. Opt. Lett..

[31] He L., Özdemir Ş.K., Zhu J., Kim W., Yang L. (2011). Detecting single viruses and nanoparticles using whispering gallery microlasers. Nat. Nanotechnol..

[32] Bradley J.D.B., Hosseini E.S., Purnawirman, Su Z., Adam T.N., Leake G., Coolbaugh D., Watts M.R. (2014). Monolithic erbium-and ytterbium-doped microring lasers on silicon chips. Opt. Express.

[33] Su Z., Li N., Magden E.S., Byrd M., Purnawirman, Adam T.N., Leake G., Coolbaugh D., Bradley J.D.B., Watts M.R. (2016). Ultra-compact and low-threshold thulium microcavity laser monolithically integrated on silicon. Opt. Lett..

[34] Frankis H.C., Bradley J.D.B. Reactively sputtered tellurium oxide films for integrated photonics. Proceedings of the Photonics North.

[35] Honma T., Ito N., Komatsu T., Dimitrov V. (2010). Thermo-optic properties and electronic polarizability in alkali tellurite glasses. J. Am. Ceram. Soc..

[36] Yoshie T., Tang L., Su S.Y. (2011). Optical microcavity: Sensing down to single molecules and atoms. Sensors.

[37] Xu D.-X., Vachon M., Densmore A., Ma R., Delâge A., Janz S., Lapointe J., Li Y., Lopinski G., Zhang D. (2010). Label-free biosensor array based on silicon-on-insulator ring resonators addressed using a WDM approach. Opt. Lett..

[38] Su Z., Li N., Frankis H.C., Magden E.S., Adam T.N., Leake G., Coolbaugh D., Bradley J.D.B., Watts M.R. (2018). High-Q-factor Al_2_O_3_ micro-trench cavities integrated with silicon nitride waveguides on silicon. Opt. Express.

[39] Pietralunga S.M., Lanata M., Ferè M., Piccinin D., Cusmai G., Torregiani M., Martinelli M. (2008). High-contrast waveguides in sputtered pure TeO_2_ glass thin films. Opt. Express.

[40] Vasiliev A., Malik A., Muneeb M., Kuyken B., Baets R., Roelkens G. (2016). On-chip mid-infrared photothermal spectroscopy using suspended silicon-on-insulator microring resonators. ACS Sens..

